# Costa Rican consumer perceptions of gene-editing

**DOI:** 10.1016/j.heliyon.2023.e19173

**Published:** 2023-08-16

**Authors:** Diego Maximiliano Macall, Johnny Madrigal-Pana, Stuart J. Smyth, Andrés Gatica Arias

**Affiliations:** a Sostenipra 2021SGR 00734, Institut Ciència i Tecnologia Ambientals (ICTA-UAB), MdM Unit of Excellence (CEX2019-000940-M), Universitat Autònoma de Barcelona, 08193 Barcelona, Spain; b University of Costa Rica, Statistics School, San José, Costa Rica; c Department of Agricultural and Resource Economics, University of Saskatchewan, 51 Campus Drive Saskatoon, Saskatchewan S7N 5A8, Canada; d University of Costa Rica, Biology School, San José, Costa Rica

**Keywords:** Biotechnology, Central America, Consumers, CRISPR/Cas9, Perception

## Abstract

Costa Rica's rice production, a large determinant of the country's food security, is being negatively impacted by frequently increasing periods of intense drought. Costa Rican scientists have applied CRISPR/Cas9 to develop drought resistant rice varieties they believe the country's rice producers could benefit from. However, would Costa Ricans consume gene edited rice or products derived from this crop? A three-part, 26-question survey administered in-person to 1096 Costa Ricans uncovers their attitudes, knowledge, and perceptions of gene editing technology and crops. Multiple regressions were built where the independent variables were age, gender, education level, and subjective economic situation. No statistically significant relationships were found in the regression coefficients. Moreover, the k-means procedure (cluster analysis) was used to categorize respondents according to their attitudes on the consumption of gene-edited foods: negative, neutral, and positive. Results show that overall, Costa Rican consumers are open to the application of gene editing in agriculture and would consider consuming products derived from the application of the technology. They are also open to gene editing technology being used to address human and animal health issues. However, Costa Rican consumers are not open to gene editing being used to “design” human traits. This study adds evidence to the emerging literature on the acceptance of gene-edited food. It also highlights the importance of informing societies of just how vulnerable agriculture, and therefore food security, is to the increasingly adverse effects of climate change.

## 1 Introduction

The Central American Dry Corridor (CADC) is the name given to a sub-region within the Central American isthmus whose climate is characterized by a marked period of intense drought that alternates with one of intense rain [1]. The CADC's geographical boundaries vary from year to year due to the influence of several climatic phenomena such as the Caribbean Low-Level Jet, and the El Niño-Southern Oscillation [[2], [3], [4]]. Over the last two decades the CADC, especially its drought component, has negatively impacted Central American food security, caused income loss, and has also spurred migration both within and across the countries it spans [5]. Drought exacerbates social problems throughout the CADC countries because half of all people living in this region live in poverty. Two-thirds of the CADC's rural population live in poverty, and an approximate two-thirds of them have inadequate nutrition [6]. According to various climate models, the agricultural sectors of these countries will be, to varying degrees, negatively impacted by increasing and more intense periods of drought [2,[7], [8], [9]]. Increasing weather variability means that these countries will need to adopt agricultural technologies to ensure that food insecurity does not increase.

Costa Rica's northwestern province of Guanacaste, where most of the country's rice production takes place, is within the CADC and routinely goes through periods of severe and prolonged drought [10,11]. In response to increasingly adverse conditions for the production of this staple crop, a group of Costa Rican scientists applied the genome editing^1^ (GE) technique clustered regularly interspaced short palindromic repeats in association with the endonuclease Cas9 (CRISPR/Cas9), to rice varieties with the goal of developing drought tolerant lines. If drought tolerance is successfully bred into Costa Rican rice varieties, the commercialization of such varieties would be immensely beneficial to both farmers and consumers [12].

Life and social sciences experts think that GE crops will outperform their genetically modified or conventional counterparts in agronomic performance and product quality [[13], [14], [15]]. These experts also posit that GE crops pose risks to human health or the environment that are no different from the risks of conventional, non-GE crops. Moreover, GE products are beginning to come to market and represent the first of a significant expansion of innovative products that will enter the market over the next decade. In Japan, the Regional Fish Institute is selling GE puffer fish and sea bream directly to consumers through an online purchase platform [16]. Brazil has approved the commercialization of GE soybeans which have entered a seed multiplication phase and are expected to be commercially available for the 2027-28 crop year [17]. A review of GE technologies predominantly at the experimental stage identifies significant contributions to the three leading Sustainable Development Goals of reduced poverty, improved food security and increased human health [18]. However, the perspective of consumers towards GE technology and crops is crucial in determining their acceptance and commercial success.

For Costa Rica specifically, the research efforts of scientists to develop a drought-resistant rice cultivar using CRISPR/Cas9 technology might be futile if consumers are not willing to purchase and consume the resultant rice products. Through an in-person administered survey, this study identifies attitudes, knowledge, and perceptions of Costa Rican consumers towards GE technology and crops (specifically, rice and beans). Given that Costa Rican scientists are at an advanced stage with their research on CRISPR/Cas9 derived drought-resistant rice cultivars, this study focuses primarily on consumer perceptions of this crop. Beans are an important component of the average Costa Rican diet and could also be the focus of GE research and development. However, scientists are keen on understanding the reaction consumers have towards GE rice before they commit to applying CRISPR/Cas9 to develop enhanced bean cultivars. Nonetheless, the results of the two questions about a hypothetical GE bean variety are also presented in this study. The insights gained from this study are vital for the future development and adoption of GE technology and crops in Costa Rica. By understanding consumer knowledge gaps and doubts about the safety and potential benefits of GE technology and crops, educational campaigns can be more effectively tailored to address these deficits and concerns. If consumers are found to be outright against GE technology, the government, academia, and the private sector might conclude that developing and commercializing this technology is not worthwhile in Costa Rica.

Previous studies have explored consumer attitudes towards GE technology or the use of GE crops from which food is or will hypothetically be derived. Gatica-Arias [19] undertook a phone survey and showed that if there are nutritional quality and price advantages from GE crop derived foods over their conventional counterparts, Costa Rican consumers would be willing to purchase these. This study compliments the Gatica-Arias [19] study because the results presented here were derived from an in-person survey of Costa Rican consumers and finds roughly the same results. Lassoued [20] found that when Canadian consumers were asked about sustainability and GE technologies, over 50% responded positively to a range of GE sustainability factors. In Japan, Otsuka [21] shows that consumers view GE crops as less natural and more similar to genetically modified organisms. Similar to Otsuka [21], this study gleans consumer understanding and perceptions of GE technology, but in contrast to that study, consumers are presented with the option to have a less expensive and more nutritious staple crop, GE rice. Given how important rice is to their daily diet, results obtained from this study show that Costa Rican consumers are more open to GE technology and crops than Japanese consumers. Similarly, Muringai [22] shows that consumers in Canada are more trusting of GE technology than genetic modification, and that they would purchase a GE crop (potatoes in that case) if it was less expensive than a conventional option. By understanding consumer behavior, with the aid of the private sector and academia, governments can develop adequate regulatory systems to accommodate burgeoning GE technologies to maximize their benefits and minimize their risks.

Section 2 begins with an overview of the rice industry in Costa Rica and underlines its significance to the country's agricultural sector and diet. This is followed by a detailed description of the 17-year research effort undertaken to create a genetically modified rice variety that is tailored to Costa Rica's unique climatic conditions and farming needs. Also discussed is the now six-year-old research effort that has gone into the development of a GE drought-resistant rice strain. Section 3 describes the survey instrument used in this study as well as how it was administered before results are presented and discussed in Section 4. Section 5 presents the conclusion of this study, in addition to summarizing the study's key findings and insights, a limitation is identified, and future research and policy recommendations are made.

## 2 Background

### 2.1 Costa Rican rice production

**Fig. 1:**
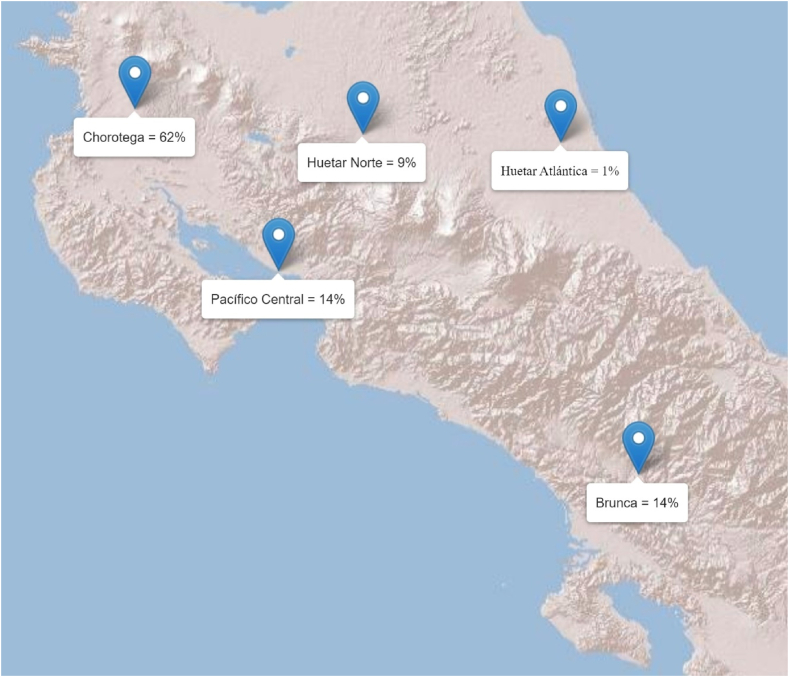
Rice production by region for the period 2020/2021.

Source: Authors based on CONARROZ [24].

The majority of rice production in Costa Rica takes place in the western socioeconomic regions of Chorotega, Brunca, and Central Pacific [23]. Among these regions, Chorotega is where most of the Costa Rican rice crop is produced (Fig. 1). Most farmers in the country (41%) produce rice on farms that are between 10 and 50 ha in size, only 6% of producers cultivate the crop on farms bigger than 200 ha [24]. In Costa Rica, 87% of all rice farms lack irrigation systems, which renders them entirely reliant on rainfall for their productivity. This susceptibility is impacting the food security of the country because the average annual per capita consumption of this staple is 53.7 kg [12,25]. In other words, every time rainfall deviates from the long-term norm (whether above or below), the food security of every Costa Rican is reduced.

**Fig. 2:**
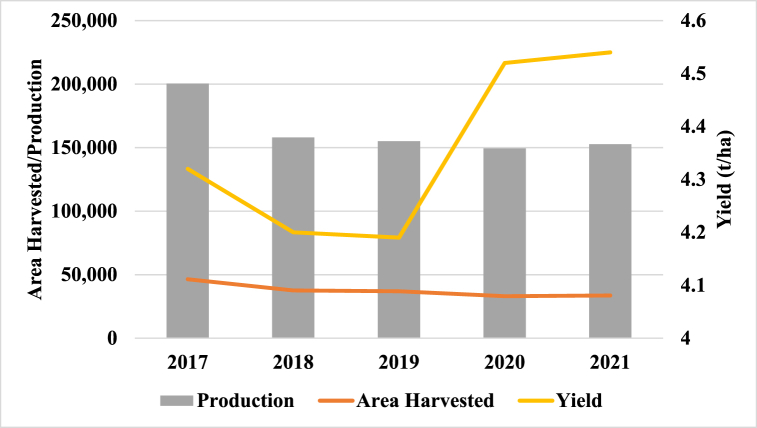
Costa Rican rice area harvested, production and yields, 2017–2021.

Source: Authors based on Costa Rican Rice Statistics [27].

According to the National Rice Corporation (CONARROZ in Spanish), between 2017 and 2021, average rice yield in Costa Rica was 4.4 t/ha (Fig. 2). Rice harvested area, as well as overall production have both decreased over this period. Conversely, over this same period, rice yield has been less predictable. After a sharp decline between 2017 and 2019, there was a sharp yield increase well past 2017 levels between 2019 and 2020. This anomalous interannual yield increase is due to high rains during the 2019–2020 agricultural year largely attributed to the hurricanes Eta and Iota [26].

### 2.2 Government intervention into the rice sector

Arroyo [28] trace and detail the four developmental stages the rice sector has gone through in Costa Rica since 1950. Between 1950 and 1965, the government's goal was to achieve rice self-sufficiency. To achieve this, the government enacted policies geared towards developing appropriate infrastructure for the crop's processing, investing money into research and development that ultimately increased rice production and quality. Between 1965 and 1975, through improved seed, producers with larger landholdings attained higher yields and small producers began to lose importance in governmental policies. Between 1975 and 1985, among other incentives, rice producers received subsidies and were given access to credit which increased both area sown and per hectare yield. From 1985 onwards, structural reforms in the agricultural sector led to a decreased governmental role in the rice sector. Initially, this spurred fluctuations in the area sown and overall production. Between 2000 and 2003, due to low international prices and with no help from the government, the Costa Rican rice sector contracted. Moreover, it was also in the early 2000s that climate events (drought and floods), and new plant pests (e.g., *Steneotarsonemus spinki Smiley*) began to negatively impact rice yields [28]. In response, in 2002 through Law N° 8285 the government created CONARROZ, which is a self-funded non-governmental organization composed of rice producers and processors whose aim is to advocate for the profitability of rice production in Costa Rica.

Currently, the Costa Rican government has once again decided to intervene in the rice sector and does so in two distinct and direct ways [28]. First, the government maintains a price control for all actors in the rice supply chain (i.e., producers, processors, retailers, and distributors). Secondly, the Costa Rican rice sector is protected with a 35% tariff on rice imports from countries outside of the Dominican Republic-Central America Free Trade Agreement. These policies are aimed at maintaining price stability in the Costa Rican rice market. However, these policies are of little help to both rice producers and consumers when there is physically no rice because of a severe drought. To maximize the efficiency of these policies, they need to be complimented with the adoption of technology in rice production itself. GE drought-resistant rice can be such a technology.

### 2.3 Rice varieties

A research group composed of members of the Center for Research in Cellular and Molecular Biology (CIBCM) and the Biology School at the University of Costa Rica, in collaboration with universities and research centers in Europe and the US, pioneered the generation of transgenic Costa Rican rice plants with the aim of conferring resistance to the white leaf virus (RHBV) and tolerance to the herbicide glufosinate. After 17 years, a RHBV and herbicide tolerant rice was obtained which in addition to an increasing productivity, also possessed culinary characteristics similar to conventional rice [29]. Research and development of novel rice varieties did not conclude at that time, as CIBCM continued with the generation of transgenic rice modified with the *Bacillus thuringiensis* vip3A gene in order to confer resistance to the *fall armyworm* (*Spodoptera frugiperda*) [30]. While the incorporation of these valuable traits into rice varieties is no small feat, they do not help farmers address the increasing threat drought poses to their rice crop.

In 2017, the same research group embarked on another project whose broad objective was to enhance rice genetics through the use of new plant breeding technologies. Specifically, the project aimed to understand whether or not the modification of the *Trehalase* enzyme active site through CRISPR/Cas9 affects rice's tolerance or resistance to salinity. Therefore, CRISPR constructs with sgRNA targeting the *Trehalase* gene (*Os10g0521000*) were introduced into rice callus by *Agrobacterium tumefaciens* genetic transformation, and 20 independents transgenic T0 lines were obtained. By amplifying selectable marker gene in the genomic DNA of T0 plantlets, 15 positive lines were identified from the regeneration plantlets and insertion and deletion of nucleotides in genomic DNA were confirmed by Sanger sequencing in six independent mutant lines (mutant frequency 40%). One plant showed a homozygous mutant, whereas the remaining 5 plants showed biallelic mutations. Currently, the selection of mutant lines against drought conditions (PEG 15%) continues. Preliminary results show that most of the PEG-selected plants survived the treatment after two months (unpublished data).

### 2.4 The need to commercialize novel rice varieties

Through various mechanisms over the past 70 years, the government has intervened to varying degrees to stabilize or expand the Costa Rican rice sector. Over the last two decades, as the climatic conditions rice farmers depend on to produce rice have deteriorated, Costa Rican scientists have collaborated with international counterparts to develop improved rice varieties. Now Costa Rican scientists are actively developing drought resistant cultivars by employing the latest generation of GE technology. However, what good are these efforts if Costa Rican consumers reject the product's derived from applying GE?

## 3 Methodology

### 3.1 Survey administration and description

To glean Costa Rican consumer attitudes, knowledge, and perceptions of GE technology and crops, a three part, 26-question survey was developed (Annex). Respondents were asked whether or not they had previously heard of GE. A factual paragraph explaining what GE is, what it could used for, and that it is not available in Costa Rica was read to respondents. Respondents were asked for their level of agreement with statements about the different uses for GE technology. The survey was developed by the cooperation of the biologists working on the development of GE drought resistant rice, the statisticians at University of Costa Rica's Statistics School, as well as the consulting of relevant literature. The questionnaire was beta tested with 100 people to ensure the questions being asked were clear, and to correct any questionnaire inconsistencies.

The survey was administered in-person in October 2018 by students from the University of Costa Rica's Statistics School to participants 18 years or older throughout the entire country.^2^ Participation in the survey was voluntary and no remuneration of any kind was given to respondents. Urban and rural proportionality was included to select the homes to which administer this survey. The surveyed sample was selected from the 2011 House Sampling Framework (Marco Muestral de Viviendas 2011 (MMV-2011)) developed by the National Institute of Statistics and Censuses (INEC in Spanish). This framework is used to undertake surveys and is managed by INEC. This framework has a total of 10,470 Sampleable Primary Units or geographical areas with clearly defined limits where all the houses in the country are located. A total of 1096 people fully answered the survey. Participants were not asked personal information beyond anonymous general socio-demographic questions. The survey was exempt of an ethics review (see Costa Rican Biomedical Research Regulatory Law N° 9234, article 25).^3^

### 3.2 Statistical analysis

Exploratory factor analysis was used to reduce the number of observed variables (dimensions) [31], and Cronbach's Alpha was employed to test the reliability of the constructed scales with values above 0.7 expected [32]. The number of dimensions in the analyses were established by characteristic values (own values) that showed a variability greater than 10%. Cluster analysis, specifically the k-means procedure [33], was used to classify respondents into three categories according to their attitudes toward consumption of GE foods: negative, neutral, or positive. Classification was designed to have the least possible variability within the constructed groups, and the highest possible variability between groups, so that each group was homogeneous. This allows for the quantification of attitudes toward the presented statements. Multiple linear regression was used to determine attitudes toward the consumption of GE foods [34]. The model included attitudes regarding the use of GE for various purposes, as well as the perceived risks and benefits associated with consuming these products. Results were analyzed using IBM SPSS Statistics Version 25 [35].

## 4 Results and discussion

### 4.1 Demographic information

Most respondents were women (52%), and overall, most people interviewed were 50 years old or older (Table 1). Of those surveyed, 25% have a university level education, 35% have a secondary education (high school diploma), and 40% have a primary education (primary school). Most people surveyed live in what is considered an urban area (74%), and the other 26% live in what is considered a rural one. The last of the demographic questions inquired about respondent income. This last question was designed to glean how difficult it is for Costa Rican consumers to earn their income, as opposed to asking what it is outright. The question was asked in this way to preserve respondent privacy, and to not discourage anyone from participating in the survey. Respondents stated that it is either difficult or very difficult (38% total) to obtain their income, while 62% of those interviewed, stated it is not that difficult for them to obtain their income, and that they are even able to put some of it into their savings. It must be noted that since most people surveyed are over 50 years old, it is not surprising that it is not that difficult for them to obtain their income because at that age it would be expected that they are well on their career paths. Moreover, according to the World Bank [36], of total Costa Ricans that could work in 2018, only 9% of them were unemployed.

**Table 1:** Costa Rican consumer demographic information.

Parameter	Percentage (%)
*Gender*^a^	
Man	48
Woman	52
*Age range*	
18–29	27
30–49	35
50 or older	38
*Education level*	
Primary or less	40
Secondary	35
University	25
*Zone in which respondent resides*	
Urban	74
Rural	26
*Income conditionality*	
Very difficult to obtain	11
Difficult to obtain	27
No major difficulty	40
I am able to save	23

a A limitation of this study is that only a binary gender choice was presented to respondents. Obtained responses may be under representative of the full spectrum of Costa Rican citizen identities.

### 4.2 Initial GE knowledge

The survey began by asking respondents how much they had previously heard or read about GE, with 96% of respondents stating that they had never heard or read anything about the topic. Similarly, in another survey gleaning attitudes towards GE, only 11% of Costa Rican university students had heard or read a little about the topic [37]. Even people at higher education institutions were not aware of this topic in Costa Rica. In another survey in Canada, the US, Austria, Germany, and Italy 45% of respondents surveyed report never having heard about GE [38], whereas in Japan 48% reported not knowing about GE applications in agriculture [39]. An indication that there may be a marked difference in knowledge of GE technologies between general populations in developed and developing countries.

After this initial question, respondents were read the following factual paragraph about GE^4^:

“Genome editing is a new technology that allows genes to be corrected in humans, plants, or animals. In the future, the possible uses of this technique could range from the improvement of crops to make them resistant to diseases, improve their productivity and nutritional profile, to the curing of diseases in humans and animals. Furthermore, it is clarified that this technique is not widespread in our country, but it is an issue that is beginning to be discussed. Would you like me to repeat what genome editing is?”

This statement set the stage for the rest of the questions in the survey. Otsuka [21] followed a similar methodology, in that consumers were first given information about GE and then asked to respond to a questionnaire about the technology. In contrast to this study, which only provided a brief paragraph on the topic, Otsuka [21] gave a 30 min lecture on GE to his sample group before proceeding to administer his survey instrument.

### 4.3 GE applications

After this statement, respondents were asked about their level of agreement with GE being used for various issues in agriculture, human health, or the environment, using a 4-point Likert scale (Table 2). Macro trends in this section of the survey emerged and can be clearly identified. For health scenarios, such as the possibility of eliminating antibiotic resistant bacteria or eliminating disease carrying mosquitoes, respondents overwhelmingly agree that GE should be applied to help address these afflictions. A sentiment echoed by consumers in Canada, the US, Austria, Germany, and Italy [38]. There was also a high level of agreement with the application of GE to save species close to extinction, or when the hypothetical application was directed at curing diseases in animals.

**Table 2:** What is your level of agreement with GE being used for the following?

Scenario	Against (%)	Neither in favor of, nor against (%)	In favor of (%)	I don't know/choose not to respond (%)
To produce climate proof food	23	2	67	8
To save animal species in danger of going extinct	14	0.6	77	8
To eliminate mosquitoes as disease vectors	10	1	82	8
To reduce the use of fertilizers in agriculture	11	2	79	9
To create animal species with different characteristics	67	3	22	9
To prevent birth defects	16	1	74	9
To produce food resistant to pests	14	0.6	77	9
To cure diseases in animals	7	2	83	9
To eliminate antibiotic resistant diseases	6	0.7	84	9
To produce food with a better nutritional profile	12	1	78	9
To solicit babies with specific characteristics like gender, eye colour, intelligence, etc.	72	0.7	18	9

Note: Against = Very against + Against; In favor of = In favor of + Very in favor of.

There was more response variability when Costa Ricans were asked about whether GE should be applied to address different issues afflicting the agricultural sector. Overwhelmingly, Costa Ricans opine that GE should be used to reduce fertilizer use in agriculture and produce more nutritious food. But when asked whether GE should be applied to ‘climate proof’ food, agreement drops to 67%. This percentage is still high, but it may be indicative that consumers are not fully aware of the extent to which climate shocks threaten food security.

When presented with scenarios in which GE is applied to human health issues, responses were in line with what has been observed in other countries and through time. Respondents think that GE should be used to avoid the inheritance of birth defects, but categorically reject its use for the creation of ‘designer babies.’ Furthermore, they were asked about the moral acceptability of using GE to reduce the risk of illnesses or birth defects in unborn babies. In addition to acceptable or unacceptable, respondents were given the option to state that they were unsure or did not know or did not have an answer. Most respondents (57%) think that the use of GE to prevent illnesses or birth defects in unborn babies is morally acceptable. Only 34% of respondents thought its use for that purpose is unacceptable.

Respondents also do not want to see GE used to create animals with ‘characteristics’ different from those of normal animals. Similarly, when questioned about the benefits of GE livestock, Japanese respondents find less benefit in them than they do in GE vegetables [40]. It seems that Costa Rican consumers, as Japanese consumers, are more accepting of green biotechnology than red biotechnology.

### 4.4 Expectations from GE applications

Furthermore, respondents expect that once GE is available in the country it will increase crop productivity, benefit animals in the country, improve the nutritional content of food, and improve the health of all Costa Ricans (Table 3).

**Table 3:** Expected benefits once GE is available in Costa Rica.

Expected benefit	Against (%)	Neither in favor of, nor against (%)	In favor of (%)	I don't know/choose not to respond (%)
Agricultural productivity will increase	14	3	68	15
Will benefit animals in the country	14.3	3	68	15
It will improve the health of Costa Ricans	15	3	68	14
The nutritional profile of food will increase	13	4	70	14

Note: Against = Very against + Against; In favor of = In favor of + Very in favor of.

Respondents were then asked about different potential risks once GE technology is available in Costa Rica (Table 4). More than half of respondents think that the risk of using the technology is either low, or none at all. Despite not being an overwhelmingly large proportion (27%), a noteworthy segment of prospective GE product consumers believe that the risks posed to human health by agricultural products derived from the technology are minimal.

**Table 4:** Perception of potential risks of GE by Costa Rican consumers.

Potential risk	None (%)	Low (%)	Medium (%)	High (%)	Don't know/No answer (%)
Risk of harming animal quality of life	19	37	4	24	16
Risk of producing environmental damage	19	4	4	25	16
Risk of agricultural products harming human health	19	35	3	28	16
Risk of negative effects on descendants	18	36	3	27	18

### 4.5 Willingness to consume a GE crop

Participants were given three scenarios about whether or not they would consume GE rice or beans (Table 5). Consumers stated that they would consume GE rice or beans if these offered a price or nutritional advantage over their conventional counterparts. A finding that is consistent with those of other studies that present consumers with the option of a biotech food with nutritional and economic advantages, over conventional counterparts and options [22,41,42].

**Table 5:** Scenario under which Costa Ricans would consume GE rice or beans.

Scenario	Yes	No	Don't know/No answer
If they were available in the country	52	38	10
If the price was the same to conventional rice or beans	65	26	9
If the nutritional value was better than conventional rice or beans	70	21	9

### 4.6 The future of GE technology in Costa Rica

A final two-part question closed the survey. The first part asked how the respondent felt about the GE technology issue? Secondly, they were presented with the options of feeling enthusiastic, cautiously optimistic, worried, or pessimistic about GE. Most respondents (34%) stated that they felt cautiously optimistic about GE, 27% reported feeling worried about it, 15% did not have an opinion, 14% felt enthusiastic about it, while only 10% felt pessimistic about the topic.

The six scales built on attitudes towards GE present acceptable reliability values since all are greater than 70%, except for the one related to genetic transformation. In addition, the mean of the scales, whose values range from 3 to 7, show intermediate values. The lowest value is presented by the scale of attitude towards genetic transformation (mean of 3) and the highest is contained in the attitude towards genetic improvement (mean of 7) (Table 6).

**Table 6:** Statistics of the scales on attitudes towards GE in different area.


Scales	Average^a^	Cronbach's Alpha (%)
Attitude towards genetic transformation	3	64
Attitude towards genetic improvement	7	85
Perception towards benefits of gene editing	7	84
Perception of risk in the use of gene editing	5	88
Gene editing consumer attitude	7	90
Attitude towards the development of gene editing research	6	75

a The scales are in a range from 0 (no support) to 10 (highest support).

The attitude towards GE agricultural product consumption was used to classify Costa Rican consumers into three groups. One group is characterized by the fact that the intensity of the scale is null, which means that they do not support the consumption of GE agricultural products (29% of the sample). The neutral group, whose mean is 1.7 and represents 20% of those who answered the survey. Lastly, the group that most supports the consumption of agricultural products showed an average of 3 and is just over half of the population (52%) (Table 7).

**Table 7:** Costa Rican consumer attitude towards the consumption of GE agricultural products.

Consumption level	Low	Neutral	High	Total
Average	0.0	1.7	3	2
Total people	938	639	1689	3267
Percentage of total	29	20	51	100

Notes: The classification of the Costa Rican consumers was carried out through conglomerate analysis, k-means procedure.

## 5 Conclusion

Costa Rica is currently enduring the initial effects of climate change, which are expected to become more acute as this century progresses. Climate change's initial effects are reducing the productivity of the crop Costa Rican food security rests on i.e., rice. In anticipation, Costa Rican scientists are working steadfastly on developing drought tolerant rice varieties using the latest GE plant breeding technology, so that the rice sector has the necessary tools to address the worsening climate crisis. However, the effort to guarantee food security will be futile if Costa Ricans are not willing to consume GE rice and its derived products. This study gauged Costa Rican consumer attitudes, knowledge, and perceptions towards GE technology and crops. Results show that Costa Rican consumers would consume GE rice if it were available in the country. Just as other studies find, consumers are open to GE being used to produce healthier food that offers nutritional and economic advantages over conventional counterparts and options. A limitation of this study is that the option presented to consumers is the crop that serves as the basis for the national diet, results might differ if a different crop or crops are presented as options such as a GE vegetable.

This study's results show that consumers are more likely to accept the application of biotechnology, GE in this case, to a health issue than an agricultural issue (‘climate proof food’ in Table 2). Costa Ricans particularly, are more accepting of employing GE to save a species from extinction and curing animal diseases, than employing the technology to future proof food. This might indicate that they are more open to addressing directly observable issues such as health issues in both humans and animals, than agriculture which they do not observe directly (74% of respondents live in an urban area). That is, given their lack of understanding of the challenges Costa Rican agriculture is going through right now, they did not support the climate proofing of agriculture as much as its application to human and animal health issues. Future research could look into whether Costa Rican consumers understand the challenges agriculture is going through, and then asking them again to weigh on the application of GE to agriculture and health issues (human and animal). Results from this survey could serve as the basis on which appropriate regulations, labeling policies, and education efforts to inform Costa Rican consumers about GE foods are built. Additionally, a campaign to inform Costa Ricans about the importance of agriculture's productivity and how climate change threatens it would also be beneficial because they might better understand why adopting technologies in this sector is crucial.

## Declarations

### Author contribution statement

Diego Macall: Analyzed and interpreted the data; Wrote the paper.

Johny Madrigal: Conceived and designed the experiments; Performed the experiments; Analyzed and interpreted the data.

Stuart Smyth: Analyzed and interpreted the data; Wrote the paper.

Andrés Gatica: Conceived and designed the experiments; Performed the experiments; Wrote the paper.

### 5.1 Data availability statement

Data will be made available on request.

### Additional information

No additional information is available for this paper.

## Declaration of competing interest

The authors declare that they have no known competing financial interests or personal relationships that could have appeared to influence the work reported in this paper.

## Figures and Tables

**Table:** 

UNIVERSITY OF COSTA RICA STATISTICS SCHOOL					A1 *# Questionnaire (Office Use)*
*A2 Complete UPM Code:___ ___ ___ ___ ___ ___ ___ ___ A3 ZONE___ A4 #Household___ ____*					
A5	*VISIT*	1	2	3	4
A6	***Day and Month***	**__ __/__ __**	**__ __/__ __**	**__ __/__ __**	**__ __/__ __**
A7	***Hour (24 H)***	**__ __: __ __**	**__ __: __ __**	**__ __: __ __**	**__ __: __ __**
A8	RESULT*	**__**	**__**	**__**	**__**
*1=COMPLETA, 2=INCOMPLETA, 3=RECHAZADA, 4=PENDIENTE, 5=NO ELEGIBLE, 6=NO REALIZADA POR OTRAS RAZONES					

Good Morning/Afternoon/Evening: My name is … …I am visiting you from the University of Costa Rica Statistics School. As part of a University project we are undertaking a survey throughout Costa Rica and I would like to interview a person who resides in this house that is Costa Rican, 18 years or older, and that is the nearest to their next birthday. *Interviewer: Once you make contact with the right person, repeat the introduction excluding the sentence in bold letters. Also add: … I would be really thankful for your cooperation. The interview is Anonymous, voluntary, and I would not take too much of your time.* *May I interview your?*	
**Interviewed Person's Name:**	***Exact time interview begins*** H ***_____ M _____ (24 Hour Format)***

	II. Attitudes about Gene Editing
EG1	How much have you heard about gene editing? 1. Nothing 2. A Little 3. Somewhat 4. A Lot ***Read the Following Paragraph to all respondents:*** Genome editing is a new technology that allows genes to be corrected in humans, plants, or animals. In the future, the possible uses of this technique could range from the improvement of crops to make them resistant to diseases, improve their productivity and national profile, to the curing of diseases in humans and animals. Furthermore, it is clarified that this technique is not widespread in our country, but it is an issue that is beginning to be discussed. Would you like me to repeat what genome editing is?

	*Hand over Card 1.* What is your level of agreement with GE being used for the following?	Against (%)	Neither in favor of, nor against (%)	In favor of (%)	I don't know/choose not to respond (%)
EG2	To produce climate proof food	1	2	3	4
EG3	To save animal species in danger of going extinct	1	2	3	4
EG4	To eliminate mosquitoes as disease vectors	1	2	3	4
EG5	To reduce the use of fertilizers in agriculture	1	2	3	4
EG6	To create animal species with different characteristics	1	2	3	4
EG7	To avoid a baby inherit a birth defect	1	2	3	4
EG8	To produce food resistant to pests	1	2	3	4
EG9	To cure diseases in animals	1	2	3	4
EG10	To eliminate antibiotic resistant diseases	1	2	3	4
EG11	To produce food with a better nutritional profile	1	2	3	4
EG12	To solicit babies with specific characteristics like gender, eye colour, intelligence, etc.	1	2	3	4

	*Continue with Card 1. Though gene editing is still not available in Costa Rica,* *How much do you agree with the following statements ….*	Against (%)	Neither in favor of, nor against (%)	In favor of (%)	I don't know/choose not to respond (%)	
EG13	Agricultural productivity will increase	1	2	3	4	
EG14	Will benefit animals in the country	1	2	3	4	
EG15	It will improve the health of Costa Ricans	1	2	3	4	
EG16	The nutritional profile of food will increase	1	2	3	4	
	When the technique is available in the country do you believe … The risk that it will damage the environment in the country will be none, low, medium, high, don't know/No answer	None (%)	Low (%)	Medium (%)	High (%)	Don't know/No answer (%)
EG17	Risk of harming animal quality of life	1	2	3	4	9
EG18	Risk of producing environmental damage	1	2	3	4	9
EG19	Risk of agricultural products harming human health	1	2	3	4	9
EG20	Risk of negative effects on descendants	1	2	3	4	9

	Would you consume gene edited rice or beans if they ….	Yes	NO	Don't Know/No Answer
EG21	… were available in the country?	1	2	9
EG22	… if the nutritional profile of beans or rice was better than their conventional counterparts?	1	2	9
EG23	… if the Price were the same as that of conventional rice and beans?	1	2	9
EG24	If you were expecting a child and gene editing reduced the risk of severe diseases throughout its life, is this something you would want used on your child to avoid these diseases? 1. Yes 2. No 9. **Don't Know/No Answer**			
EG25	Do you think that using gene editing so that babies have less of a risk of diseases or other maladies is morally acceptable or inacceptable? 1. Acceptable 2. Inacceptable 3. Its Not Safe 9. **Don't Know/No Answer**			
EG26	***Hand over Card 2*.** How do you fell with this topic of gene editing? Would you say you feel enthusiastic, cautiously optimistic, worried or pessimistic? 1. Pessimistic 2. Worried 3. Neutral 4. Cautiously Optimistic 5. Enthusiastic 9. **Don't Know/No Answer**			

## Footnotes

1 GE is used throughout the paper to refer to ‘gene editing’ or ‘gene edited.’

2 Students were instructed in all the stages related to administering a survey: planning, administering, data recollection, digitization of questionnaires, inconsistency corrections, and results presentation among others.

3 See Article 25 of the Biomedical Research Regulatory Law N° 9234. http://www.pgrweb.go.cr/scij/Busqueda/Normativa/Normas/nrm_texto_completo.aspx?param1=NRTC&nValor1=1&nValor2=77070&nValor3=96424&strTipM=TC.

4 The paragraph has been translated from Spanish to English by Diego M. Macall.
